# Development of Cells Repository of *Betta* Species: A Tool for Genetic Conservation and Biotechnological Advancement

**DOI:** 10.3390/ani16030408

**Published:** 2026-01-28

**Authors:** Sukumal Prukudom, Yanika Piyasanti, Santi Poungcharean, Suparat Chaipipat, Kornkanok Sritabtim, Juthathip Jurutha, Chonphoom Phan-poe, Rungthiwa Sinsiri, Soranuth Sirisuay, Kannika Siripattarapravat

**Affiliations:** 1 Kasetsart University Veterinary Diagnostic Laboratory-Bangkhen, Faculty of Veterinary Medicine, Kasetsart University, Bangkok 10900, Thailand; sukumal.pru@ku.th (S.P.); yanika.piy@ku.th (Y.P.); suparat.chaip@ku.th (S.C.); kornkanok.sr@ku.th (K.S.); juthathip.ju@ku.th (J.J.); chonphoom11@kuvacb.com (C.P.-p.); fvetrws@ku.ac.th (R.S.); 2 Department of Anatomy, Faculty of Veterinary Medicine, Kasetsart University, Bangkok 10900, Thailand; 3 Department of Fishery Biology, Faculty of Fisheries, Kasetsart University, Bangkok 10900, Thailand; ffisstpr@ku.ac.th; 4 Department of Aquaculture, Faculty of Fisheries, Kasetsart University, Bangkok 10900, Thailand; ffissns@ku.ac.th; 5 Department of Pathology, Faculty of Veterinary Medicine, Kasetsart University, Bangkok 10900, Thailand

**Keywords:** wild betta, type locality, cell culture, cryopreservation

## Abstract

Thailand harbors a remarkable diversity of betta (Siamese fighting fish); however, their wild populations are threatened by habitat loss and genetic pollution from released ornamental fish. Therefore, effective conservation strategies are needed to extend traditional in situ and ex situ methods, which are resource-intensive and prone to external threats. This study established a practical approach for cultivating cells from ornamental bettas and applying it to 11 wild species collected from their specific type localities in Thailand. Additionally, relevant available data on water properties were collected. Cell isolation was achieved from larval caudal fin bud and adult caudal fin tissues, with larval specimens exhibiting a significantly higher isolation success rate (83.3%) and a shorter initial cultivation period (13.27 ± 3.2 days) compared to adult cells (38.8% success, 24.33 ± 7.8 days initial period). After overcoming senescence, the cell doubling time (DT) was approximately 2 days, and cryopreservation assays demonstrated post-thaw viability over 80%, ensuring the long-term viability. While larval isolation is challenging, adult fin biopsy provides a superior alternative by bypassing breeding and euthanasia. This non-lethal, repeatable sampling method allows for precise species identification via external morphology, offering a highly efficient strategy for conservation. Cell isolation was achieved for 10 of the 11 species, 9 of which resulted in established, cryopreserved cell lines, representing the first maintained cell lines from larval and adult tissues of *Betta* species and providing a novel, valuable resource for both biotechnology and the crucial biodiversity conservation of this important freshwater fish of Thailand.

## Simple Summary

Siamese fighting fish (*Betta* spp.), Thailand’s national aquatic animal, is world-famous for its vibrant colors and unique behaviors. However, many wild species in Thailand are now threatened, creating an urgent need for effective conservation. In this study, we developed a practical method to collect and preserve cells of the *Betta* species using ultra-low temperature storage (cryopreservation) for long-term protection. We successfully isolated cells from eleven different wild species while also analyzing the water quality of their natural habitats. A key advantage of our approach is its versatility; we can successfully collect and culture cells from various sources and across different life stages, from young larvae to adult fish. Crucially, our results prove that these cells remain viable and healthy following prolonged storage. Beyond serving as a genetic reservoir for Thailand’s betta diversity, these established cell lines provide a powerful tool for researchers. They allow us to study how bettas remarkably adapt to changing environments without needing to disturb live fish from the wild. This work offers a sustainable path to safeguard the future of these iconic fish.

## 1. Introduction

Bettas, commonly known as the Siamese fighting fish, are native freshwater fish found in Southeast Asia. Five breeds of bubble-nesting bettas and seven breeds of mouth-brooding bettas have been discovered in Thailand, highlighting the country’s rich betta biodiversity [1]. Over centuries, bettas have been domesticated and selectively bred, resulting in an ornamental strain whose individuals are renowned for their vibrant colors and striking fin patterns. Their relatively short lifespan of 1–2 years necessitates continuous breeding and sales for pet replacement. The economic trends of betta farming fluctuate with market demand, with shifts in popularity leading to the decline of some less desirable ornamental strains [2]. In contrast to the diverse appearances of their ornamental descendants, wild bettas are typically smaller, with shorter fins and a brownish-green coloration. Common habitats for these wild are shallow, slow-moving waters such as ditches, rice paddies, and muddy ponds.

Natural habitats are being threatened increasingly by pollution, urbanization, and rising temperatures. Climate change severely impacts aquatic life, with global warming potentially leading to a 40% loss of fish species with a 3.2 °C increase [3]. Since bettas live in lentic water systems, they are particularly vulnerable to these changes, which could lead to genetic alterations [4]. Documentation of wild bettas in Thailand began over 150 years ago [5]; however, the status of their original type localities is now largely unknown. Therefore, a comprehensive reassessment of wild bettas is urgently needed, along with the establishment of living collections to preserve their genetic diversity.

Bettas are valuable animal models for addressing biological questions. Their vibrant coloration and diverse fin morphology are used for studying molecular mechanisms [6], while their aggressive behavior makes them ideal for behavioral science [7] and hormone study [8]. Cellular-level biological exploration in bettas remains a promising area of research. While nearly 300 fish cell lines are listed in international repositories such as the American Type Culture Collection (ATCC) and European Collection of Authenticated Cell Cultures (ECACC) since 1962 [9], permanent somatic cell lines from bettas have not been documented. Fish cell lines have been isolated successfully from various tissues, including fins [10], the liver [11], brains [12], and the heart and swim bladder [13]. However, fins and larvae are the most frequently used tissue samples [9]. Notably, in vitro cell isolation has been reported from *B. splendens* skin chromatophores, demonstrating their utility as cytosensors in response to pathogenic bacteria [14] and as a neurotransmitter [15], and highlighting the crucial role of establishing a cell line of *Betta* species, which would serve as both a biobank and a valuable tool for diverse biological research [16].

In this study, we collected 11 species of wild bettas from their type localities, as well as analyzed the water properties at each site. We developed cell cultivation and cryopreservation protocols using ornamental bettas, evaluating their growth and post-storage viability. Then, these validated methods were applied to wild betta specimens from different life stages to establish a viable cell cryorepository. Reliable and viable cell lines are an invaluable resource for biodiversity conservation and genomic research. These cell lines offer a potential pathway for restoring declining wild betta populations through somatic cell nuclear transfer (SCNT), or reproductive cloning, thereby enabling the sustainable preservation of valuable broodstock and the revival of species facing biodiversity depletion.

## 2. Materials and Methods

### 2.1. Sources of Ornamental and Wild Bettas

The ornamental fish sample, a “halfmoon tail betta” (a bubble-nesting crossbred betta), was purchased from the Chatuchak Ornamental Fish Market, Bangkok, Thailand. The broodstocks of wild bettas were collected from sites that were either close to the type specimen’s locality characteristics or matched the description of the type specimen. In summary, five bubble-nesting and six mouth-brooding betta species were collected following population surveys in 11 provinces across four different regions of Thailand. *Betta pi*, the 12th wild betta species, was identified in Narathiwat province, Thailand. However, data for this species remain unavailable at present, owing to its low population numbers, the inability to acquire adult tissue or larval samples due to the specimen’s delicate condition, and unsuccessful breeding attempts.

### 2.2. Water Parameter Analysis of Habitats

Water properties at most wild betta collection sites were assessed. The dissolved oxygen level, temperature, and salinity were measured using a dissolved oxygen meter (YSI Pro2030, Yellow Springs, OH, USA). A pH meter (EcoSense100, Yellow Springs, OH, USA) was used to record the water pH. Water hardness was analyzed according to Grasshoff [17] and American Public Health Association [18]. Wild-caught animals were transported and acclimatized in a husbandry unit prior to taxonomic assessment and subsequently were used in a cell isolation process. The bettas were subjected to comprehensive taxonomic evaluation to verify their identification according to previously reported literature (Supplementary Data), ensuring consistency with type localities and habitat characteristics through the analysis of meristic (fin count), morphometric (body measurement), and qualitative (pigmentation) characteristics.

### 2.3. Animal Husbandry and Breeding Management

The husbandry of all betta specimens was conducted at the Faculty of Fisheries, Kasetsart University. Adult fish were maintained for a 1–2 month acclimatization period prior to the experiments. Animal health was rigorously monitored based on physical condition and behavioral indicators, including anorexia (refusal to feed for >3 days), persistent lethargy, and respiratory distress (rapid opercular movement or surface gasping). Bettas considered unfit for anesthesia, due to poor health or suboptimal body condition, were excluded from the fin biopsy procedure. The healthy broodstock samples of the bubble-nesting bettas, including the halfmoon tail bettas, were acclimatized separately in 500 mL glass bottles. Environmental conditions were routinely monitored, with a water temperature of 27–28 °C, 3–5 mL/L of dissolved oxygen, and a pH of 6.5–7.5. The broodstock were fed twice daily with live adult brine shrimps (*Artemia salina*). A pair of broodstock was mated by placing them in a 20 × 25 × 30 cm glass container and separating them with a clear plastic panel. Emergent plants (*Ipomoea aquatica*) were provided as a substrate for males to build a bubble-nest. The ready-to-brood pairs were detected by the male’s nest-making behavior and a change in body coloration. Then, the clear plastic panel was removed and the pair usually spawned within 1–2 days. After spawning, the female was immediately removed to prevent her from eating the eggs, and the male was allowed to provide continuous care for the fertilized eggs.

For the mouth-brooding bettas, 4–5 broodstock pairs were acclimatized and kept in a glass container measuring 30 cm × 90 cm × 30 cm. Submerged shrubs (*Anubias* spp.) and polyvinylchloride (PVC) pipes (10 cm in length) were provided as shelter. The fish were fed twice daily with adult brine shrimps (*Artemia salina*) and grindal worms (*Enchytraeus buchholzi*). The fully mature broodstock mated during the nighttime, and the resulting mouth-brooding males were observed the next morning. These males could be distinguished from non-brooding males by their bulging buccal membrane, refusal to eat, and habit of hiding in the provided plants or PVC pipes. The fertilized eggs gradually developed, hatching and being released from their father’s mouth within 10–12 days (typically 11 days). During this period, there was no interference from the parental males to prevent the premature release or devouring of the eggs. After hatching, the mouth-brooding males were isolated to avoid cannibalism. Upon their release, the offspring were fed with water fleas (*Moina macrocopa*) and brine shrimp larvae.

### 2.4. Culture Medium for Fish Cell Cultivation

LDF-NAC culture medium was modified from established zebrafish culture medium protocols [19,20]. The LDF stock medium was composed of Leibovitz L15 (Gibco, Waltham, MA, USA, cat. no. 41300-039), Ham’s F12 (Gibco, cat. no. 21700-75), and DMEM (Gibco, cat. no. 12800-017) in a ratio of 50:35:15 (*v*/*v*/*v*). To make LDF-NAC medium, LDF stock medium was supplemented with 2 mM N-acetyl-L-cysteine (Sigma-Aldrich, St. Louis, MO, USA, cat. no. 8199), 1 mM ascorbate-2-phosphate (Sigma-Aldrich, cat. no. A8960), 1 mg/mL bovine insulin (Sigma-Aldrich, cat. no. I6634), 10 mM HEPES (Sigma-Aldrich, cat. no. 15630-080), 1% *v*/*v* of antibiotic–antimycotic (100X) (Thermo Fisher Scientific, Waltham, MA, USA, cat. no. 15240062), 5% *v*/*v* fetal bovine serum (Hyclone, Logan, UT, USA, Thermo Fisher Scientific, at. no. 10091148), and 1% *v*/*v* trout serum.

### 2.5. Larval Tissue Preparation and Cell Isolation

**Figure 1:**
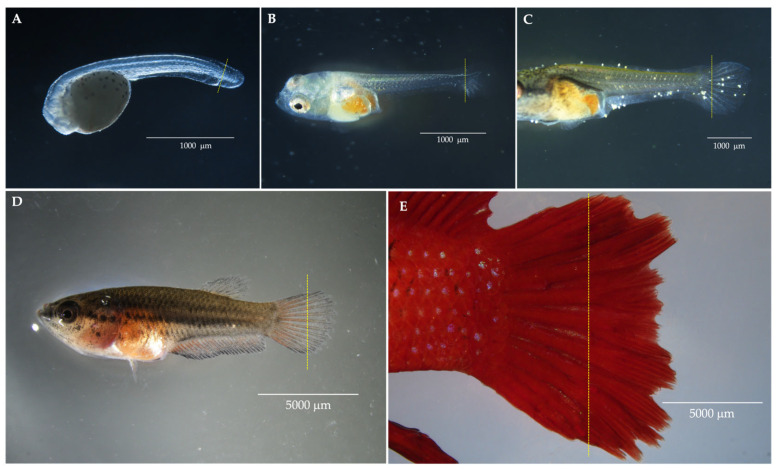
Illustration of tissue biopsy process from caudal fin of betta at various developmental stages. A yellow vertical line represents an incision site. Panels (**A**–**C**) show collection from caudal fin bud of yolk-sac larva (**A**); pre-larva (**B**); and post-larva (**C**). Panels (**D**,**E**) illustrate the biopsy of approximately one-third of the caudal fin from juvenile (**D**); and adult (**E**) bettas.

Tissue collections were strictly timed according to specific embryonic developmental stages. Larval tissue collections were carried out at the Faculty of Veterinary Medicine, Kasetsart University. Morphologically normal larvae were euthanized with 0.04% *w*/*v* tricaine until heartbeat cessation, then washed in sterile embryo medium [21] with 100 parts per million (ppm) of methylene blue. Chorions were manually removed, and the larvae were washed again in sterile embryo medium with 1% *v*/*v* antibiotic–antimycotic (100X). Caudal fin buds were excised using a #11 scalpel. Samples from the wild bettas were obtained from several developmental stages (yolk-sac larval, pre-larval, post-larval stages, and juvenile), as shown in Figure 1A–D. Decontamination involved incubation in 0.01–0.05% *v*/*v* sodium hypochlorite for 1 min, followed by three rinses (each for 3 min) in 1% *v*/*v* antibiotic–antimycotic (100X) in sterile Hanks’ Balanced Salt Solution (HBSS). Tissue dissociation was performed in a biosafety cabinet by chopping caudal fin buds in LDF stock medium with a #11 scalpel, followed by repeated pipetting. For larvae >2 day-post-fertilization (dpf), tissue was incubated in warmed TrypLE™ Express Enzyme (Thermo Fisher Scientific, Waltham, MA, USA) at 28 °C for 1–2 min, then the reaction was stopped with twice the volume of LDF-NAC medium, followed by centrifugation at 1200× *g* round per minute (rpm) for 5 min. The cell pellet was resuspended in 200–400 µL LDF-NAC medium and transferred to collagen-coated 96-well or 48-well plates for incubation at 28 °C.

### 2.6. Adult Tissue Preparation and Cell Isolation

For adult specimens, biopsies were performed following a standard acclimatization period, with each individual subjected to tissue collection only once to ensure data independence and animal welfare. Adult tissue biopsies were performed at the Faculty of Fisheries. Mature, fully grown bettas were anesthetized with 2% *v*/*v* clove oil until they were immobilized, with reduced respiration and no tactile response. Then, each fish was rinsed and placed on a sterile petri dish. Caudal fins were rinsed with sterile embryo medium containing 1% *v*/*v* antibiotic–antimycotic (100X), and one-third was excised using a #24 scalpel (Figure 1E). Fin tissue was transferred to sterile HBSS with 100 ppm methylene blue. Each fish was monitored for full recovery in clean water, which was confirmed by an increased respiratory rate, mobilization, and restored equilibrium, before being returned to the husbandry site. For decontamination, fin tissue was vortexed three times (each for 3 min) in sterile HBSS with 100 ppm methylene blue, then repeated the process with sterile HBSS with 1% *v*/*v* antibiotic–antimycotic (100X). The tissue was bleached with 0.07% *v*/*v* sodium hypochlorite by vortexing for 3 min, followed by three washes in sterile HBSS with 1% *v*/*v* antibiotic–antimycotic (100X). Dissociation in a biosafety cabinet involved transferring fin tissue to 100 IU/mL collagenase in 5 mL LDF stock medium supplemented with 0.05 mL HEPES, shaken at 290 rpm for 40–60 min at 28 °C. An equal volume of LDF-NAC was added, followed by centrifugation at 1200 rpm for 5 min. The cell pellet was resuspended in 2 mL LDF-NAC and transferred to a collagen-coated 25 cm^2^ flask for incubation at 28 °C.

### 2.7. Cell Culture and Propagation

Larval and adult tissue specimens were randomly assigned to either pooled or individual cultivation. The study was designed to minimize bias by ensuring that tissue excision and cell isolation were performed by separate researchers who were blinded to each other’s procedures. Larval cells were maintained individually in 96-well plate or pooled in 48-well plate. After 48 h of cell attachment, 50% of the medium was replaced with LDF-NAC. For adult cells, which were grown in flasks, growth was initially restricted by tilting the flask. Then, 50% of the medium was replaced until the cells covered approximately 25% of the surface. Later, the flask was laid flat with 4 mL of medium. Both larval and adult cells were subcultured at approximately 70% confluence. Cells were rinsed with sterile HBSS. Then, they were treated with 28 °C-warmed 100 IU/mL collagenase in LDF-NAC for 8 min, followed by 28 °C-warmed TrypLE™ Express Enzyme for 5 min. After this, twice the volume of LDF-NAC medium was added. The cell suspension was centrifuged at 1200 rpm for 5 min, the supernatant was removed, and the pellet was resuspended in LDF-NAC. Viable cells were counted using trypan blue exclusion assay before reseeding. The primary cells, hereafter referred to as cells of *Betta* species, were observed and imaged using an Olympus IX71 microscope (Hachioji, Japan) with a Nikon DS-Fi3 camera (Minato, Japan) and the NIS-Elements software version 5.42.01.

### 2.8. Cryopreservation and Cell Viability Assessment

To cryopreserve cells of *Betta* species, they were enzymatically detached and resuspended in a cryoprotective medium containing 10% *v*/*v* dimethyl sulfoxide in fetal bovine serum and then transferred to cryogenic vials (Corning, Corning, NY, USA, cat. no. 430488). The cells were frozen at −80 °C overnight, then stored in a liquid nitrogen dewar. Viability was evaluated at intervals up to 12 months. The evaluation commenced with rapidly thawing each vial in a 28 °C-water bath. Then, the thawed suspension was transferred to 9 mL of LDF-NAC medium and centrifuged at 1200 rpm at 4 °C for 5 min to remove the cryoprotective medium. Cell viability was assessed using trypan blue exclusion assay (Gibco, cat. no. 15250061) and LIVE/DEAD™ Viability/Cytotoxicity Kit (Thermo Fisher Scientific, cat. no. L3224). Both assays were used to evaluate the percentage of live cells, following the manufacturer’s instructions. The cell mixture was loaded into a hemocytometer, and the live cell ratio was assessed using an Olympus IX71 fluorescent microscope.

### 2.9. Immunofluorescent Assay

Cells of *Betta* species were fixed with 4% *v*/*v* paraformaldehyde (PFA) in phosphate-buffered saline (PBS[[-]) for 10 min at room temperature. Then, they were washed for 30 min with PBST (0.1% *v*/*v* Triton X-100 in PBS[[-]). The cells were permeabilized with 0.5% PBST (0.5% *v*/*v* Triton X-100 in PBS[[-]) for 15 min, followed by another 15-min wash with PBST. Next, the cells were incubated with a blocking solution (5% *v*/*v* goat serum in PBST, Sigma-Aldrich, cat. no. G9023) for 30 min, followed by a 15-min wash with PBST. The primary antibody (rabbit anti-fibronectin [human], Sigma-Aldrich, cat. no. F3648) was applied and incubated overnight at 4 °C, after which the cells were washed with PBST for 30 min. Then, the secondary antibody (goat anti-rabbit IgG, Alexa Fluor 568; Invitrogen, Carlsbad, CA, USA, cat. no. A-11011) was incubated for 90 min. Following a 30-min wash with PBST, the cells were counterstained with 500 µL of Hoechst 33,342 solution (10 mg/mL, Sigma-Aldrich, cat. no. 14533) for 10 min. After another 15-min wash with PBST, SlowFade^TM^ Gold Antifade Mountant (Thermo Fisher Scientific, cat. no. S36936) was added to the well. Images were acquired using an Olympus IX71 fluorescent microscope and an Optikam HDMI camera (Montreal, QC, Canada) controlled using the OptikaISview software, version 3.9.0.

### 2.10. Chromosome Spread Preparation

For chromosome spreading, cells of *Betta* species were cultured at approximately 60–70% confluence, ensuring a minimum yield of 2 × 10^6^ cells. The cells were arrested in metaphase by adding 10 μL/mL of Gibco KaryMAX^®^ Colcemid™ Solution (Gibco, cat. no. 15212012) in LDF-NAC medium, followed by incubation for 45 min at 28 °C. Following enzymatic dissociation using 28 °C-warmed 100 IU/mL collagenase in LDF-NAC for 8 min, followed by 28 °C-warmed TrypLE™ Express Enzyme for 5 min, the resulting single-cell suspension was centrifuged at 1200 rmp for 5 min; the supernatant was discarded. The cell pellet was subjected to a hypotonic treatment by slow, dropwise addition of a pre-warmed 0.56% *w/v* potassium chloride hypotonic solution to a 5 mL final volume, which was then incubated for 20 min at 37 °C. After a subsequent centrifugation, the cells were fixed by resuspending the pellet in a freshly prepared fixative (acetic acid:methanol, 1:3 ratio). This fixation step involved a 5-min incubation at room temperature and centrifugation at 1200 rmp for 10 min, and was repeated twice. The final cell pellet was resuspended in 100−200 μL fixative. The fixed cells were then dropped vertically onto dry, clean glassslides (one drop per slide) from a suitable height to ensure optimal cell rupture and chromosome spreading. Finally, the slides were stained with 10% *v/v* Giemsa, and metaphase spreads were examined and counted under a light microscope at 1000X magnification to determine the karyotype.

### 2.11. Statistical Analysis

Isolation success rates were compared between larval and adult cells of *Betta* species using a chi-squared test. One-way ANOVA was conducted to analyze differences in average initiation periods (days of passage 0–passage 1) among the four sample groups: individual caudal fin bud; pooled caudal fin bud; individual caudal fin; pooled caudal fin. Post-thaw viability of cells was assessed across storage durations using one-way ANOVA. Results were presented as mean ± standard deviation values. The normality of the data distribution was assessed using the Shapiro–Wilk test prior to performing the ANOVA. Linear regression models were used to evaluate the slopes of cumulative population doubling levels (CPDL) between two sample categories. All statistical analyses were performed at the 95% confidence level with a significance threshold of *p* ≤ 0.05 using GraphPad Prism version 10.2.1 (339).

## 3. Results

### 3.1. Survey and Collection of Wild Bettas from Type Localities with Concurrent Water Parameter Assessment

**Figure 2:**
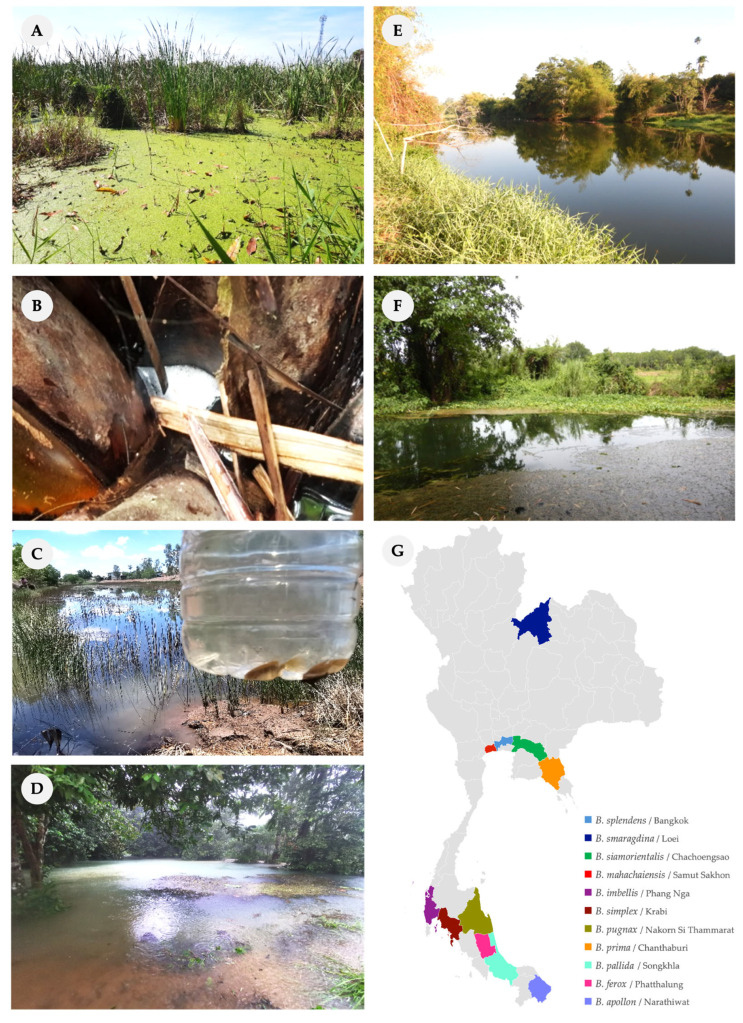
Geographical distribution (**G**) and some landscape photographs of collected wild betta sites during surveillance, displayed by species and provinces. Images of sampling locations are illustrative of specific species of bettas: Narrow-leaf cattail swamp—*B. splendens* (**A**); Nipa palm brackish swamp—*B. mahachaiensis* (**B**); Ground chestnut swamp and paddy field—*B. siamorientalis* (**C**); Pond connected with river on limestone geology—*B. simplex* (**D**); Bank of canal—*B. prima* (**E**); Vegetated river—*B. smaragdina* (**F**).

We collected wild betta specimens from the type localities of nine species to preserve their original lineages. For *B. smaragdina* and *B. ferox*, whose type localities are outside Thailand, specimens were collected from locations reported by local communities. In Thailand, bubble-nesting bettas are found in the Central, Eastern, Northeastern, and Southern regions, often in water sources near human settlements such as rice paddies and rubber plantations. Mouth-brooding bettas (except for the *B. prima* found in the Eastern region) are primarily distributed in the Southern region, inhabiting mountain-adjacent habitats such as rivers on limestone geology and hill streams, as well as brackish water (Figure 2).

**Figure 3:**
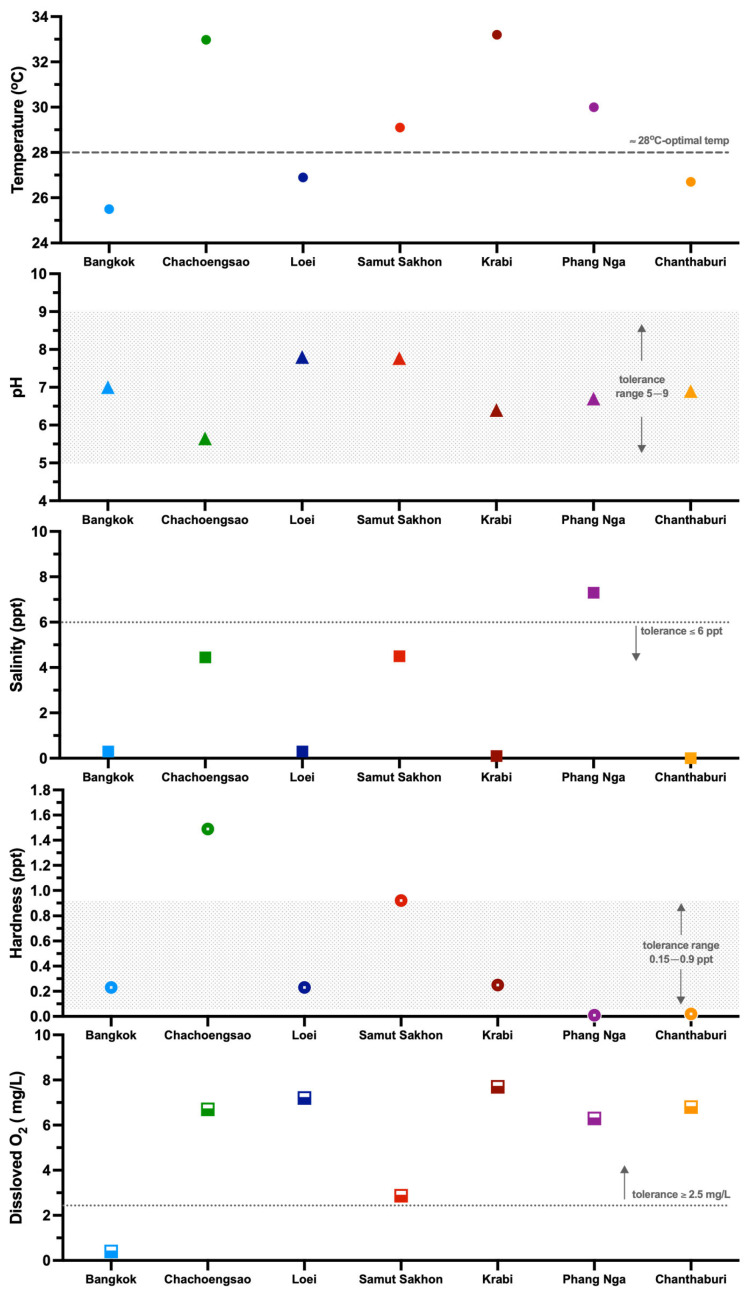
Graphs of water parameters (temperature, pH, salinity, hardness, and dissolved oxygen level) of habitat ecosystems in Bangkok, Chachoengsao, Loei, Samut Sakhon, Krabi, Phang Nga, and Chanthaburi provinces. Temp = temperature, ppt = part per thousand.

Water quality data were recorded from five habitats: Bangkok, Loei, Chachoengsao, Samut Sakhon, and Chanthaburi (Figure 3). Water quality data of the Phang Nga, Krabi, Nakorn Si Thammarat, Songkhla, Phatthalung, and Narathiwat sites were not accessible because fish samples were collected by local individuals due to private land access and security restrictions. The water temperatures varied widely, from a low of 25.5 °C in Bangkok to highs of 32.98 °C and 33.2 °C in Chachoengsao and Krabi, respectively. While Loei and Chanthaburi were slightly below the optimal 28 °C [22,23], Samut Sakhon and Phang Nga had intermediate ranges (29.1–30.0 °C). All surveyed sites had a pH within the tolerable range of 5–9 [22]. Chachoengsao was the most acidic (5.65), while Loei and Samut Sakhon were the most alkaline (pH 7.77–7.8). Other sites had a near-neutral pH. Water hardness and salinity varied substantially by location. While most sites had low hardness and salinity, Chachoengsao emerged as an outlier with hardness levels of 1.49 parts per thousand (ppt), surpassing the typical 0.9 ppt threshold, alongside a salinity of 4.45 ppt, which is notably elevated relative to standard values [24]. Notably, Phang Nga had very soft water (0.012 ppt) but the highest salinity (7.3 ppt), exceeding the tolerance limit [22]. Dissolved oxygen levels were generally above the 2.5 mg/L tolerance limit [25], except for Bangkok (0.4 mg/L) and Samut Sakhon (2.87 mg/L). Diverse environmental conditions—including varied temperatures, pH, and salinity (from freshwater to brackish), along with a wide range of dissolved oxygen levels—likely exert considerable selective pressures on wild betta populations. These pressures likely influenced their distribution, survival, and physiological adaptations, potentially leading to species that uniquely adapted to challenging conditions such as low oxygen or high salinity.

### 3.2. Successful Isolation of Cells from Adult and Larval Tissue of Betta Species

Cryopreservation of cells is a recommended method for sustainably maintaining betta biodiversity [26]. The current study investigated the isolation and in vitro cultivation of cells from both larval and adult ornamental bettas (Table 1) with the goal of applying the findings to wild species. The samples were categorized into individual and pooled groups. Individual samples, representing the smallest quantity for cultivation (preferably biodiversity representatives), were examined for their isolation success rate, defined as the number of samples successfully subcultured from passage 0 (P0) to passage 1 (P1), and the initial cultivation period (the time from P0 to P1). This comparison helps to determine the most effective method for preserving genetic diversity. The percentages of successful isolations from individual larval caudal fin buds of 1-, 2-, and 3-dpf larvae were all 78.5% (11/14). All pooled samples from larval caudal fin buds were isolated successfully from larvae aged at 1- and 2-dpf (4/4). For adult tissue origin, isolation was achieved in 35.7% (15/42) of individual caudal fins and 57.1% (4/7) of pooled caudal fins. The overall isolation success rate of larval cells (83.3%) was significantly higher (*p* ≤ 0.05) than that of adult cells (38.8%). Initial cultivation periods were calculated based on the time elapsed between the date of first isolation (P0) and the date of first subcultivation (P1). The average initial periods for betta larval cells were 13.27 ± 3.2 days for individual samples (*n* = 11) and 15.25 ± 3.2 days for pooled samples (*n* = 4). For betta adult cells, the average initial periods were 24.33 ± 7.8 days for individual samples (*n* = 15) and 16.25 ± 9.5 days for pooled samples (*n* = 4). Individual adult cells had significantly longer initial cultivation periods than individual larval cells (*p* ≤ 0.05). However, no significant differences were observed among other groups (*p* > 0.05), as shown in Table 1.

### 3.3. Characteristics of Cells of Betta Species During In Vitro Propagation

**Figure 4:**
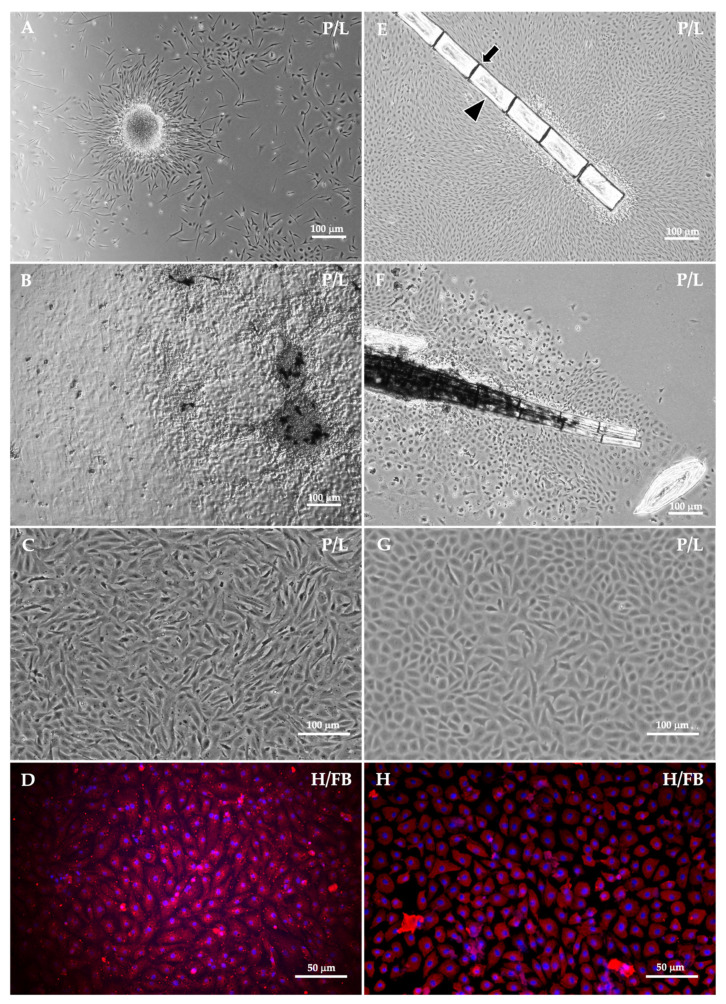
Primary cultures of cells of *Betta* species showing various morphologies. Larval cells outgrowth from larval tissues ((**A**,**B**), 40X). Adult cells explanted from scales and fin ray segments ((**E**,**F**), 40X); arrowhead indicates hemisegment and arrow indicates intersegmental joint of fin ray (**E**). Subsequently, cells of *Betta* species were subcultured and appeared more uniform in both larval cells ((**C**), passage 13, 100X) and adult cells ((**G**), passage 61, 100X). Larval ((**D**), passage 7) and adult ((**H**), passage 49) cells of *Betta* species were positive for fibronectin protein (red, 200X), whereas nuclei of cells stained with Hoechst 33,342 (blue, 200X). *p*/L: phase-contrast; H: Hoechst 33,342; FB: fibronectin.

**Figure 5:**
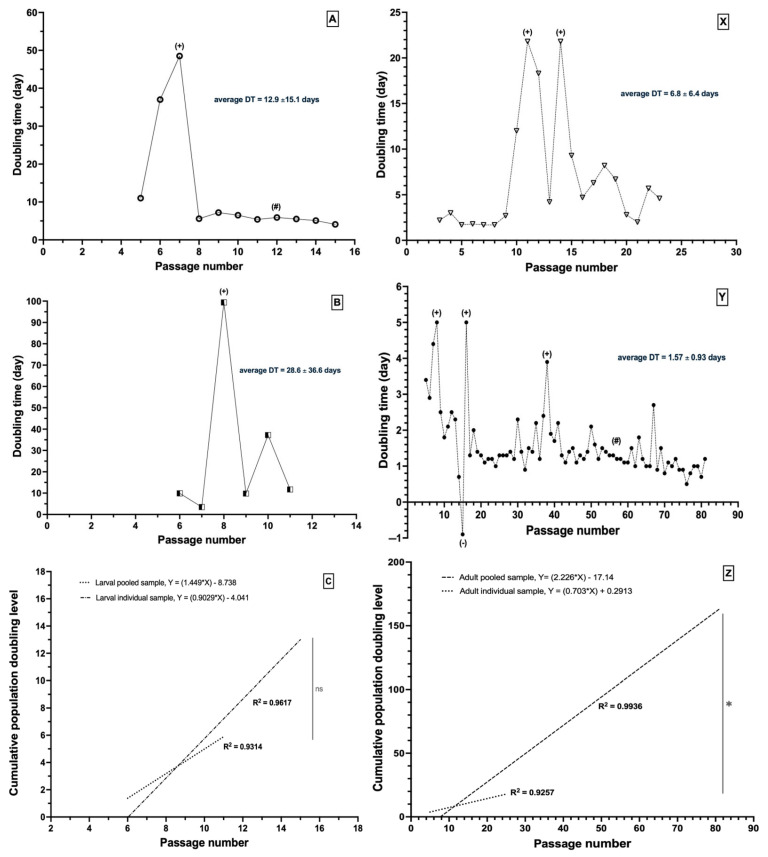
Doubling time of cells of *Betta* species demonstrated the variation of proliferative patterns among larval cells (individual (**A**) and pooled (**B**)) and adult cells (individual (**X**) and pooled (**Y**)), indicating remarkably extended DT (+), negative DT (-), and consistent DT (#). Cumulative population doubling level (CPDL) plots demonstrated a strong correlation with passage number (**C**,**Z**); data were analyzed using a linear regression model. The slopes between individual and pooled samples of larval cell lines (**C**) were not significantly different (ns, *p* = 0.0911), while the slopes between individual and pooled samples of adult cell lines (**Z**) were significantly different (*, *p* < 0.0001). ns = non-significance.

During the primary culture, small larval fragments served as explants for cell outgrowth and produced a variety of cell types (Figure 4A,B). Over time, fibroblast-like cells gradually became the dominant population. Following several passages, the explants mostly dislodged, and the cell morphology became more uniform (Figure 4C), transitioning from a heterogeneous primary culture to a more homogeneous population dominated by fibroblast-like cells. These cells were characterized by consistent spindle-shaped morphology and uniform dimensions throughout the subsequent passages, allowing for accurate cell counting. The doubling time (DT), the amount of time it takes for a cell population to double in number, was calculated to monitor cell growth. The larval cells had diverse growth patterns (Figure 5A,B). In most cases, the initial DT was within 1–2 weeks. However, after several passages, we observed a notably extended DT (indicated with “+” in Figure 5A,B), suggesting a replicative senescence. The average DT of representative larval cells was 12.9 ± 15.1 days and 28.6 ± 36.6 days for individual and pooled samples, respectively. Later, individual larval cells had a relatively consistent doubling time (indicated with “#” in Figure 5A), indicating their potential for long-term cultivation. The cumulative population doubling level (CPDL) indicates the cumulative number of cell replicative cycles over time [27]. The linear graph of larval cells had a relatively high coefficient of determination (R^2^) of representative individual (R = 0.9617) and pooled (R = 0.9314) larval cells indicatong a relatively high correlation between passage number and the CPDL (Figure 5C); however, there were no significant differences between the CPDL slopes of individual and pooled samples (*p* = 0.0911).

In the adult cell culture, the scattered scales and fin ray segments served as explants for cell outgrowth (Figure 4E,F). The initial cell population was a mix of epithelial-like and fibroblast-like cells; however, the epithelial-like cells eventually became dominant and formed confluent clusters (Figure 4G). The DT plots of the adult cells had a variety of growth patterns. All samples had an extended DT, either with single or multiple events (indicated with “+” in Figure 5X,Y), or a negative DT (indicated with “−” in Figure 5Y) when the number of harvested cells was less than the initial seeding number. The mean DT of the representative line was 6.8 ± 6.4 days for the individual adult samples. The pooled adult cells exhibited the capacity for long-term cultivation, with a mean DT of 1.57 ± 0.93 days, and this cell line reached over 60 passages. The CPDL plots of the representative individual (R = 0.9257) and pooled (R = 0.9936) adult cell lines had a high R^2^ value. Despite this, the linear slopes of the CPDL plots were significantly different (*p* ≤ 0.0001) among individual and pooled adult cell lines (Figure 5Z).

**Figure 6:**
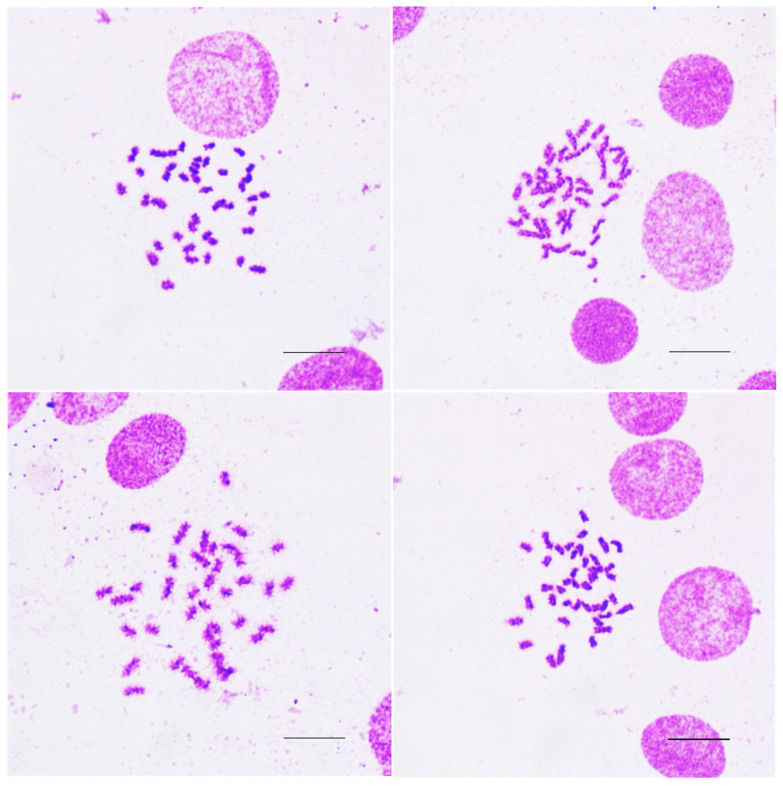
Metaphase chromosome spreads of cultured cells (passage 64, 1000X) derived from caudal fin tissue of adult ornamental betta. Cells have normal diploid chromosome number (2n = 42). Scale bar = 10 μm.

Immunofluorescent assays were performed in larval cells at passage 7 (Figure 4D) and in adult cells at passage 49 (Figure 4H). Representative cell lines derived from both larvae and adults exhibited uniform fibronectin expression, indicating a homogeneous population of fibroblast-like cells. Metaphase spreads of caudal fin-derived cells from adult ornamental betta showed a normal diploid chromosome number (2n = 42) [28] after being cultured through 64 passages (Figure 6).

### 3.4. Viability of Cell of Betta Species Following Cryopreservation

**Figure 7:**
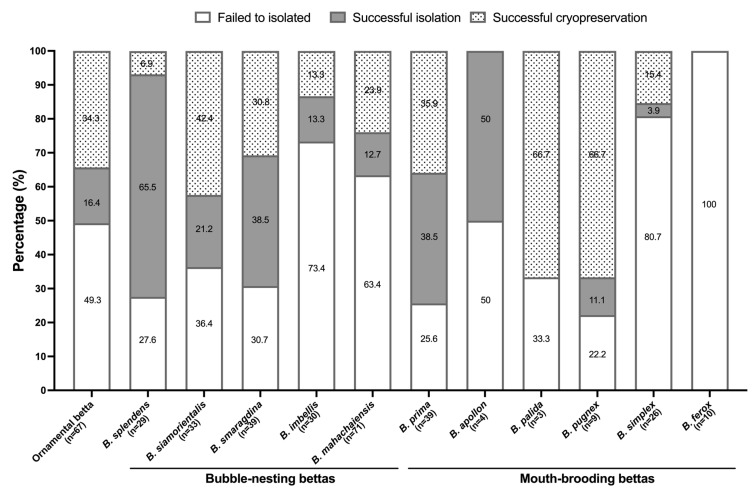
Percentage of isolation and cryopreservation success of five species of bubble-nesting bettas and six species of mouth-brooding bettas, derived from field surveillance in Thailand. Summarized results from ornamental betta-derived cells included in the figure, with percentage of isolation failure (white bar) and remainder representing isolation success (grey bar combined with dotted bar).

**Figure 8:**
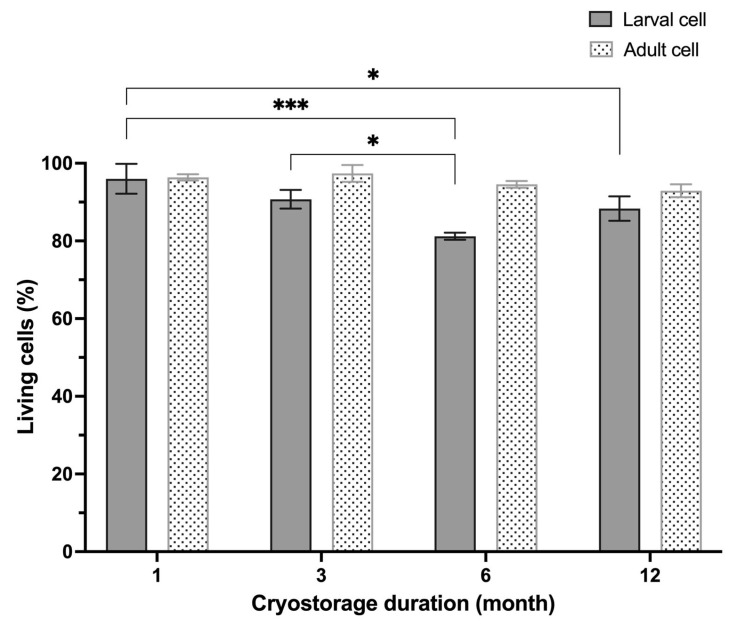
Post-thaw viability of larva- and adult-derived cells of *Betta* species evaluated by LIVE/DEAD™ kit over 12 months of cryopreservation. Different symbols (*, ***) indicate significant differences in the percentage of viable cells across storage durations.

Successful bioresource banking is not only limited to the primary cultivation, but also to the post-thaw survival of the cryopreserved cells for future utilization. In this study, both larval and adult cells of the *Betta* species were collectively passaged and cryopreserved. Cultured lines derived from both larval and adult tissue of ornamental betta were cryopreserved successfully (34.3%, *n* = 23, Figure 7). For post-thaw cell viability assessment, cells of the *Betta* species were cryopreserved simultaneously, with cell revivals performed chronologically at 1, 3, 6, and 12 months (Figure 8). Triplicate vials were thawed at a time, and live cell counting was repeated twice for each vial. While the viability rate of larval cell line decreased after storage for 6 (*p* ≤ 0.01) and 12 (*p* ≤ 0.05) months, the adult cell line maintained a consistent viability rate above 80% throughout the 1-year period, as evaluated by the LIVE/DEAD™ kit (Figure 8), consistent with findings from trypan blue exclusion assay (Figure S1). Following thawing, the cells underwent revival and were successfully recultivated.

### 3.5. Establishment of Frozen-Cell Repository of Wild Bettas in Thailand

**Figure 9:**
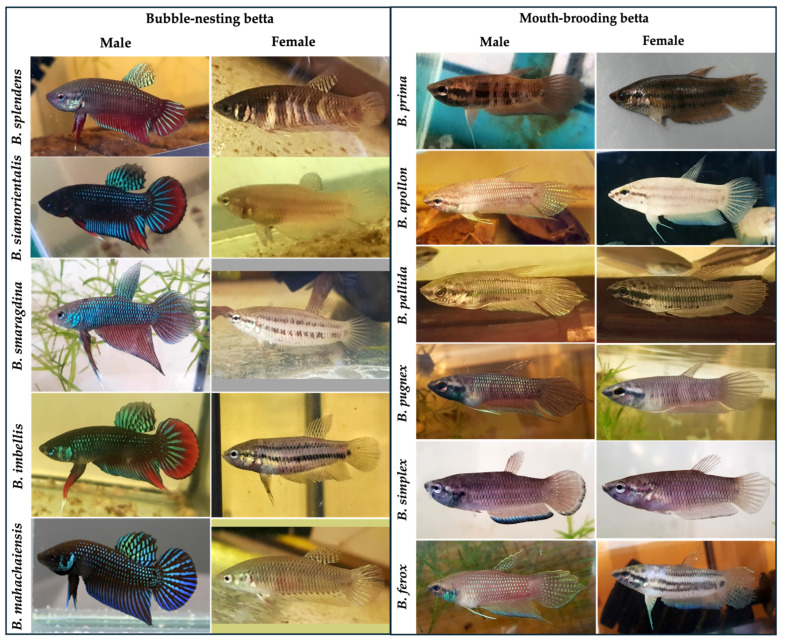
Morphological appearances of male and female wild-caught bettas. Comprehensive taxonomic evaluations were used to confirm species of bubble-nesting (*n* = 5) and mouth-brooding (*n* = 6) bettas.

Tissue specimens derived from the five species of bubble-nesting and the six species of mouth-brooding bettas (Figure 9) were harvested for cell isolation, following the previously stated protocols. Given the variable nature of field samples, they were isolated according to their preparability and potential cell yield. Isolation success rate was assessed upon the first subcultivation. As shown in Figure 7, the outcomes for wild betta were categorized into isolation failure (white bar), isolation success only (gray bar), and isolation with successful cryopreservation (dotted bar). Regarding total isolation success (grey bar combined with dotted bar), three out of five bubble-nesting betta species demonstrated high isolation success rates, exceeding 50%. These species were *B. splendens*, *B. siamorientalis*, and *B. smaragdina.* In contrast, *B. imbellis* and *B. mahachaiensis* had total isolation success rates of less than 40%. While the proportion of successfully cryopreserved cell lines was lower (dotted bar), we were nonetheless able to cryopreserve cells from all bubble-nesting bettas. Four out of six mouth-brooding bettas showed high total isolation success rates (grey bar combined with dotted bar), all exceeding 50%. These species included *B. prima*, *B. apollon*, *B. pallida*, and *B. pugnax*. On the other hand, *B. simplex* had a very low success rate of less than 20%. However, cryopreservation success (dotted bar) was achieved in only four species: *B. prima*, *B. pallida*, *B. pugnax*, and *B. simplex*. Notably, cells from *B. apollon* could not be successfully cryopreserved despite their high isolation success. The isolation of cells from *B. ferox* was entirely unsuccessful, meaning no cryopreservation could be attempted.

## 4. Discussion

Bettas are important freshwater fish in Thailand, playing a vital role in various aspects of the country’s culture, economy, and biodiversity. The fishery industry and the national bioresources management program are supported by the sustainability of wild bettas through their species diversity, as well as ornamental lines through their diverse morphologies and colorations [29]. The current study obtained 11 species of wild bettas from various sites across Thailand, with the associated water parameter measurements revealing fluctuating environmental conditions in these ecosystems. We found that most of the measured parameters had values outside the reported tolerance ranges for bettas, including temperature. Other studies have reported erythrocyte abnormalities and gill damage in *B. rubra* following exposure for 2 weeks to temperatures exceeding 32 °C [23], highlighting the vulnerability of tropical ectotherms (such as those experienced by wild bettas living near their thermal limits) to rising temperatures compared to temperate species [30].

Wild bettas have the capacity to acclimate and survive across a broad pH spectrum [22]. For example, *Betta siamorientalis* and *B. imbellis* were found living in water with extremely high hardness, which critically impacts reproductive performance [24]. However, in the current study, the rice paddy habitat of *B. siamorientalis* had high salinity, whereas the swamp habitat of *B. imbellis*, located close to rubber and palm plantations, had low salinity. This marked difference in saltiness suggests two possibilities: either wild bettas have a strong capacity to adapt to seemingly unsuitable conditions, or each species has specific environmental preferences crucial for its survival. Given their proximity to human communities, it is plausible that improper wastewater management is contributing to oxygen depletion, whether from residential or industrial activities. These low dissolved oxygen levels could create stressful or even lethal conditions for many aquatic organisms, potentially limiting their distribution or impacting their health and reproductive success in these habitats. These findings underscore the vulnerability of wild betta populations to environmental changes, particularly in the context of climate change, and emphasize the critical need for further research into their specific tolerances and adaptation mechanisms to inform conservation strategies.

Cryopreservation is an efficient method for storing living cells that can be repopulated when needed, making it an excellent tool for establishing biobanks of living genetic material for future use. Compared to the ongoing aquaculture of betta (a form of ex situ conservation) or the protection and restoration of natural habitats (a form of in situ conservation), cryopreservation using liquid nitrogen dewars requires considerably less labor and maintenance. However, currently, there is no evidence of a cell line of the *Betta* species being reported with constant propagation and successful cryopreservation.

Here, we developed protocols for isolating and cultivating cells of the *Betta* species in vitro using samples from the ornamental betta. Additionally, we investigated their growth patterns and the feasibility of ultra-low temperature preservation. Subsequently, we applied these verified protocols to wild betta obtained from field surveys. In this study, the cell culture techniques were adapted from published protocols for zebrafish cell culture studies. The LDF-NAC culture medium is a novel formulation modified from LDF medium [19], and has been used successfully to culture zebrafish cells and, in the current study, cells of the *Betta* species. A basal medium of L-15, F-12, and DMEM was applied in various ratios, as used for platy fish melanoma [31], zebrafish blastula [19], and zebrafish fin tissue [20]. L-15 is common in fish cell culture [9] due to its CO_2_ independence [32]. The LDF-NAC medium was supplemented with 1% trout serum; lower concentrations have been used in Japanese flounder [33]. Our methods were designed to accommodate all types of samples and sampling numbers that might be collected in the field. Based on our current results, cells of the *Betta* species could be successfully isolated, propagated, and cryopreserved from both larval and adult tissues. Additionally, samples could be prepared either individually or by pooling them together (2–3 combined samples).

Since mature animal tissue naturally comprises various cell types, its use as a culture source usually yields a mixed cell population characterized by diverse morphologies. The betta caudal fin has complex structures, including lepidotrichia or segmented rays (as indicated by the arrowhead in Figure 4E) and actinotrichia (a flexible fin edge), which contain dermis, epidermis, vessels, and collagenous joints (as indicated by the arrow in Figure 4E). Such anatomical complexities likely contribute to the variety of cells obtained [34,35]. Our findings reveal that the fin-derived cell lines have superior long-term growth (> 60 passages), potentially due to the fin’s regenerative capacity [36]. Actinotrichia serves as the initial site for lepidotrichia growth during both development [37] and regeneration [38]. Other studies have highlighted the role of actinotrichia in fin regeneration by either inducing lepidotrichia-forming cells (LFC) [39] or expressing *lh 1*, a gene involved in collagen I and II synthesis [40]. These findings are consistent with our observation that collagen-coated culture flasks promoted the adherence of explants. It was observed that the culture conditions in the current study potentially favored the long-term growth and survival of adult cells over those of larval cells. The fin-ray and scale fragments yielded by enzymatic dissociation served as explants, which progressively provided a source of cells for culture. Different cell origins likely influence morphology and propagation that may vary between larval and adult cells under the same conditions. Both cell types tested positive for intracellular fibronectin, a marker characterizing fibroblasts responsible for fish skin and scale regeneration [41]. This staining pattern confirms the fibroblast-like phenotype of the established cell lines of the *Betta* species.

Cell isolation success was influenced by several factors. The bubble-nesting bettas nurture eggs in a saliva nest until hatching (around 2 dpf), whereas the mouth-brooding species carry eggs in the male’s mouth to a more advanced larval stage (Figure 1B–D). This difference impacts the risk of contamination in larval cultures. Field-collected samples, especially mouth-brooders, often have varied developmental stages. Larvae ingest and excrete, which increases the contamination risk during tissue excision. Contamination risk in adult specimens, particularly in wild betta, could be correlated with habitat conditions. Furthermore, the adult fish’s thick mucosal coating could hinder effective decontamination. Our current findings indicate that larval cells showed significantly higher isolation success than adult cells. Adult cells required a longer initial cultivation period than larval cells, which could potentially increase aging and apoptosis [42], as evidenced by negative (−) and spiked doubling times (+) in adult cells (Figure 5Y). Cellular senescence takes place either through the shortening of the telomere [43] or stress-induced DNA damage and oxidative stress [44]. Once this challenge had been overcome in the current study, the doubling time stabilized at approximately 2 days, resulting in a relatively consistent cumulative population doubling level (Figure 5Y). This stability reflected a uniform cell population, further evidenced by the strong and widespread expression of fibronectin (Figure 4D–H). For future reference, we recommend that studies on isolating cells from adult tissue consider using pooled specimens. This approach was found to be more efficient, as it substantially reduced the initial cultivation period required for successful cell growth. The observation of multiple replicative crises is a phenomenon that may be encountered during the isolation of cell lines of the *Betta* species. We propose that an extended doubling time, beyond the values established in the current report, should be considered as a criterion for terminating the cell culture to optimize resource allocation and prevent unnecessary time expenditure. The growth patterns of wild betta-derived cells could not be fully analyzed due to the interference of numerous factors. Although they were harder to isolate, the wild betta-derived cells had similar or better cryopreservation success than the ornamental betta-derived cells (Figure 7). Several wild betta fin-derived cell lines were propagated successfully for over 20 passages, demonstrating robust renewal capabilities and potential as permanent cell lines.

These established cell lines are foundational to innovating ornamental fish with highly marketable phenotypes, a process made feasible through advancements in the genome editing of somatic cell lines. Beyond commercial applications, these cells offer high utility for cell-based in vitro analysis, allowing researchers to investigate the physiological adaptations of wild bettas to suboptimal environmental conditions at a cellular level. A particularly challenging application is the reprogramming of somatic cells into induced pluripotent stem cells (iPSCs), which represents a significant frontier in fish biotechnology and species preservation [45]. Furthermore, genetic analysis of these established cell lines will help address genetic contamination between wild and ornamental populations and facilitate the study of inbreeding depression, providing insight into the factors driving the decline of biological fitness in isolated populations.

Monitoring post-thaw viability indicated that while partial cell loss occurred during the freezing process, a viability rate of over 80% was successfully maintained. We recommend that the cryopreservation efficacy of cells of the *Betta* species be monitored at 3–5-year intervals to ensure long-term viability. Additionally, future studies should incorporate DNA integrity assessments for long-term cryopreserved cells, as this remains a critical factor in genetic conservation efforts.

## 5. Conclusions

Here, we successfully established a robust protocol for propagating and cryopreserving cell lines of the *Betta* species, achieving over 80% viability upon thawing, making them readily available for future applications. This method was implemented on wild species. The cryopreserved cells of the *Betta* species constitute an invaluable national genetic resource, derived from wild populations inhabiting their native environments across Thailand, encompassing documented holotype localities. The success in preserving these wild betta-derived cells ensures the sustainable existence of their biodiversity. Furthermore, the study raised the concerning issue of water quality in wild betta habitats, which is potentially impacted by both anthropogenic activities and climate change. Further investigation is required regarding the adaptive responses of wild species to suboptimal environmental conditions. Since bettas are native to Thailand, cell lines of *Betta* species are a valuable in vitro model for environmental science and toxicology, offering a way to study these fields without relying as heavily on live wild specimens and, importantly, avoiding the potential physiological discrepancies associated with using fish cell lines from different geographical regions. In vitro assays utilizing cell lines offer a high degree of experimental consistency owing to their homogenous populations. Application of cell lines of *Betta* species for in vitro assay is time- and cost-efficient, in accordance with the “3Rs” principles [46] and Organization for Economic Co-operation and Development (OECD) guidelines [47].

## Figures and Tables

**Table 1:** Cell isolation success and average initiation period of ornamental betta.

Type of Specimen	Tissue Samples—Life Stage		Cell Isolation Success		Average Initiation Period (Mean ± Standard Deviation)
			**N**	***n*** **(%)**	
Larva	Individual caudal fin bud	- 1 dpf	9	7	13.27 ± 3.2 ^a^
		- 2 dpf	3	3	
		- 3 dpf	2	1	
	Pooled caudal fin bud	- 1 dpf	2	2	15.25 ± 3.2 ^ab^
		- 2 dpf	2	2	
	Total		18	15 (83.3) *	
Adult	Individual caudal fin		42	15	24.33 ± 7.8 ^b^
	Pooled caudal fin		7	4	16.25 ± 9.5 ^ab^
	Total		49	19 (38.8) **	

Different marks (*, **) indicate significant differences (*p* ≤ 0.05) of isolation success between larval and adult samples. Different letters in “Average initiation period” column indicate significant differences (*p* ≤ 0.05) among four sample groups. Pooled sample consisted of 2–3 specimens per culture, dpf = day post fertilization, N = number of samples, *n* = number of successful isolates.

## Data Availability

The datasets used and/or analyzed during the current study are available from the corresponding author upon reasonable request. No publicly archived data were generated or analyzed in this study.

## Supplementary Materials

The following supporting information can be downloaded at: https://www.mdpi.com/article/10.3390/ani16030408/s1. Figure S1: Post-thaw viability of larva- and adult-derived cells of *Betta* spp. evaluated by trypan blue exclusion assay over 12 months of cryopreservation. Figure S2: The LIVE/DEAD™ Viability/Cytotoxicity Kit was used to assess post-thaw viability, with representative images showing (A) live and (B) dead cell staining. Geographic localities of wild betta based on previously reported literatures [48,49,50,51,52,53,54,55].

## Abbreviations

The following abbreviations are used in this manuscript:

HBSS	Hanks’ Balanced Salt Solution
DT	Doubling time
CPDL	Cumulative population doubling level
OECD	Organization for Economic Co-operation and Development
°C	Degree Celsius
