# Supplementary material for: Development of Cells Repository of Betta Species: A Tool for Genetic Conservation and Biotechnological Advancement
Source: Animals (Basel). 2026 Jan 28;16(3):408. doi: 10.3390/ani16030408 (PMC12896975; doi:10.3390/ani16030408)
Supplement: Supplementary file 1 [file animals-16-00408-s001.zip › Proofread_supplementary data.pptx]

## Slide 1
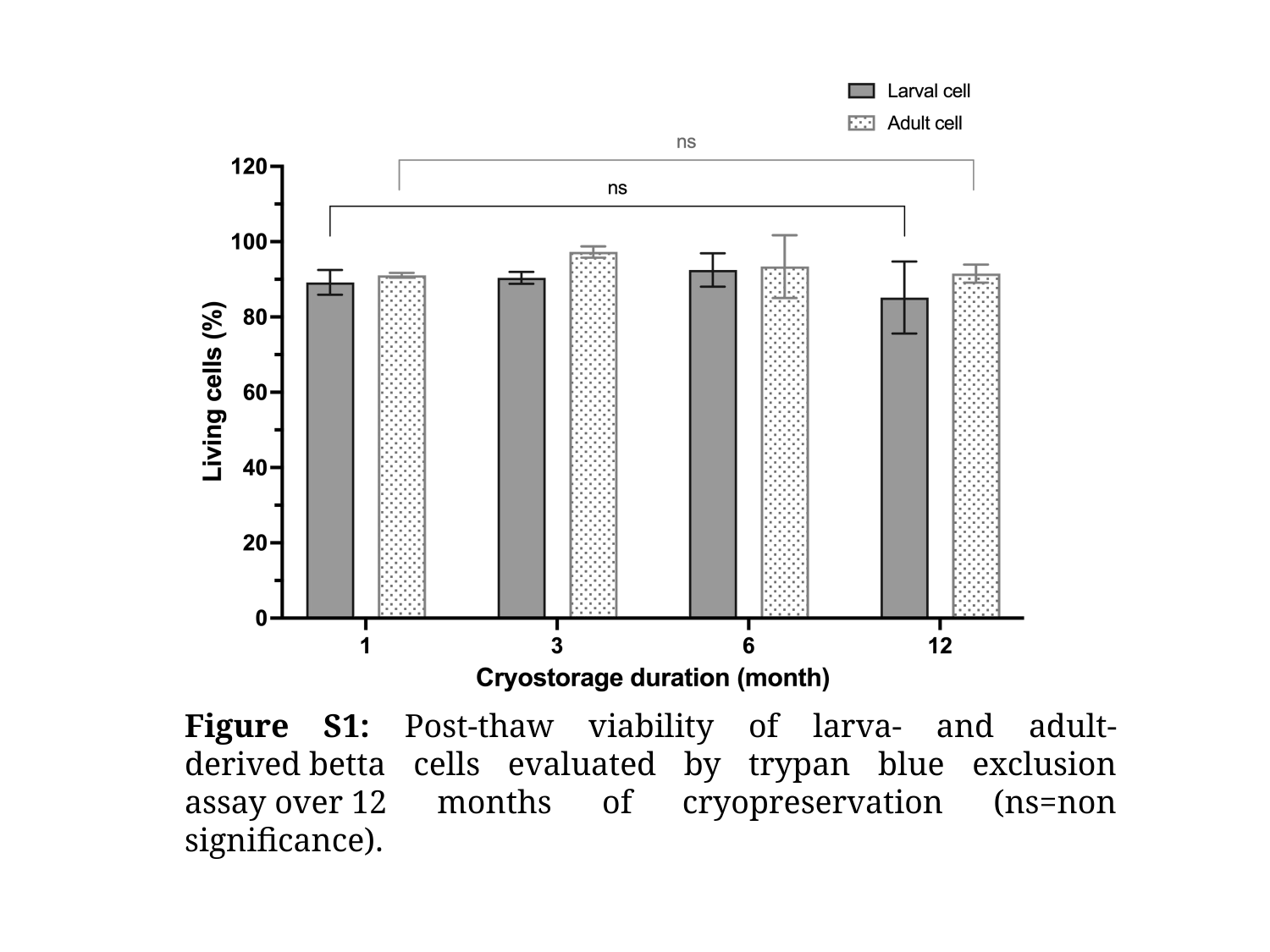

Figure S1: Post-thaw viability of larva- and adult-derived betta cells evaluated by trypan blue exclusion assay over 12 months of cryopreservation (ns=non significance).

## Slide 2
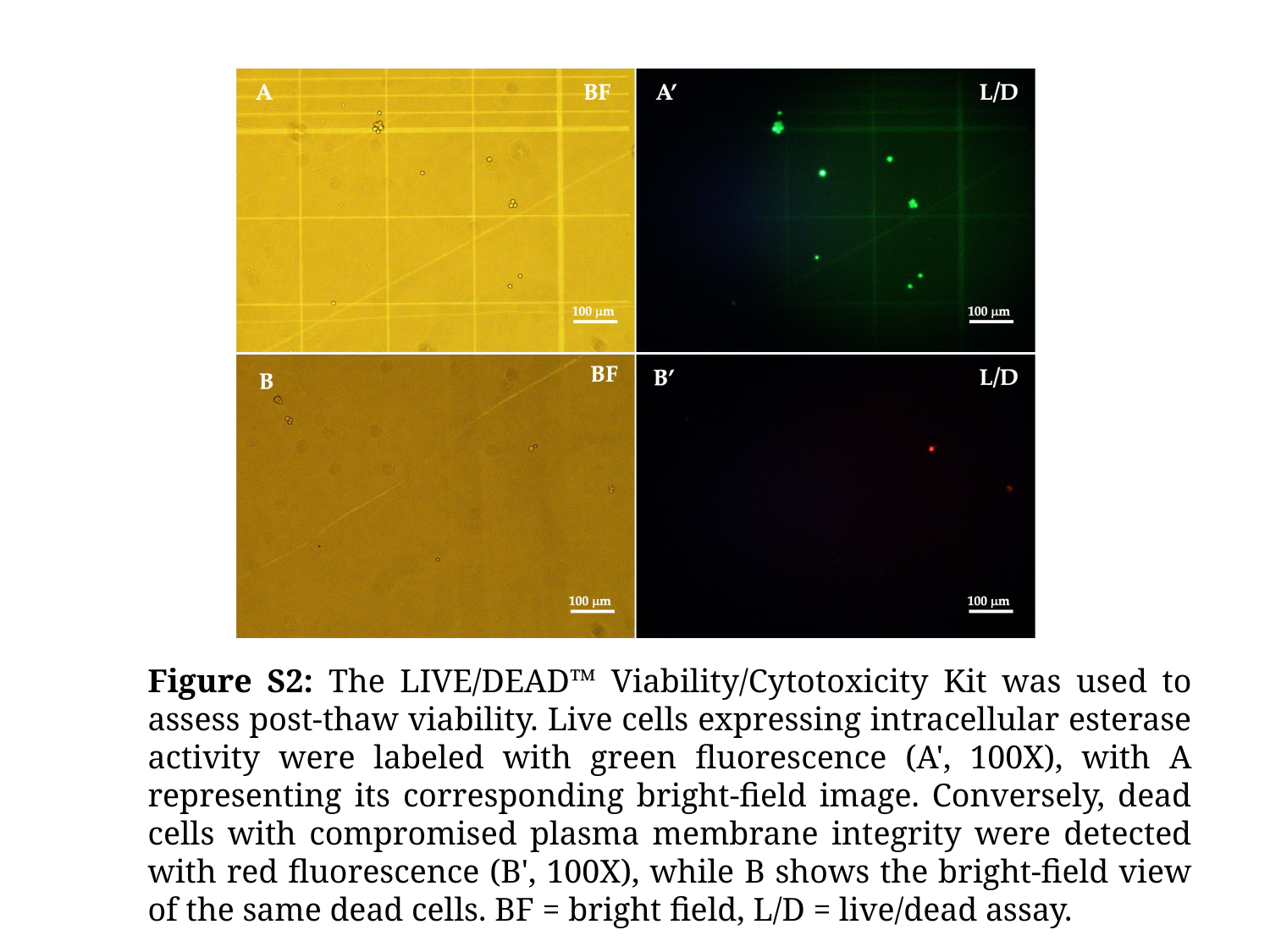

Figure S2: The LIVE/DEAD™ Viability/Cytotoxicity Kit was used to assess post-thaw viability. Live cells expressing intracellular esterase activity were labeled with green fluorescence (A', 100X), with A representing its corresponding bright-field image. Conversely, dead cells with compromised plasma membrane integrity were detected with red fluorescence (B', 100X), while B shows the bright-field view of the same dead cells. BF = bright field, L/D = live/dead assay.

## Slide 3
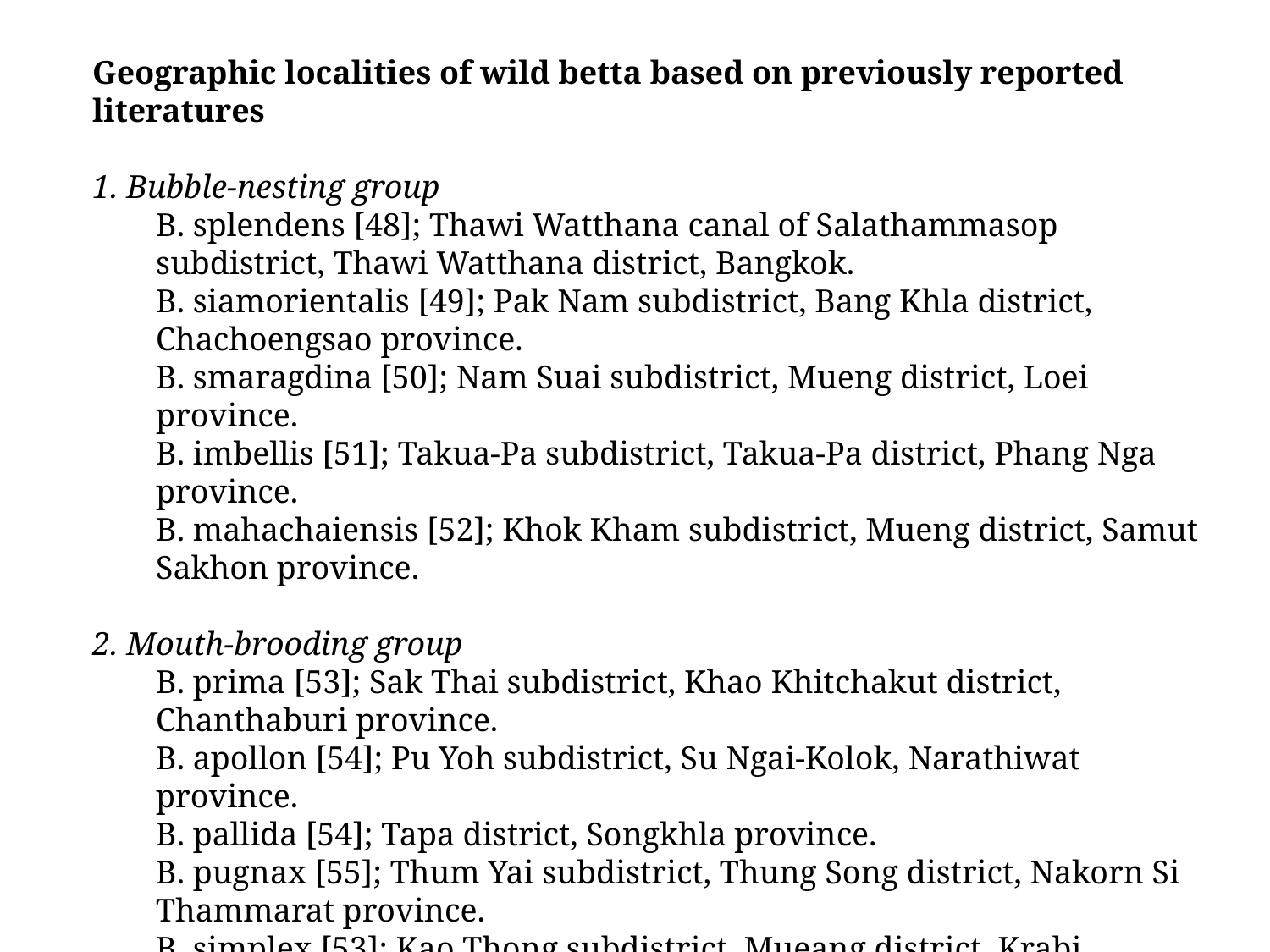

Geographic localities of wild betta based on previously reported literatures
1. Bubble-nesting group
B. splendens [48]; Thawi Watthana canal of Salathammasop subdistrict, Thawi Watthana district, Bangkok.
B. siamorientalis [49]; Pak Nam subdistrict, Bang Khla district, Chachoengsao province.
B. smaragdina [50]; Nam Suai subdistrict, Mueng district, Loei province.
B. imbellis [51]; Takua-Pa subdistrict, Takua-Pa district, Phang Nga province.
B. mahachaiensis [52]; Khok Kham subdistrict, Mueng district, Samut Sakhon province.
2. Mouth-brooding group
B. prima [53]; Sak Thai subdistrict, Khao Khitchakut district, Chanthaburi province.
B. apollon [54]; Pu Yoh subdistrict, Su Ngai-Kolok, Narathiwat province.
B. pallida [54]; Tapa district, Songkhla province.
B. pugnax [55]; Thum Yai subdistrict, Thung Song district, Nakorn Si Thammarat province.
B. simplex [53]; Kao Thong subdistrict, Mueang district, Krabi province.
B. ferox [54]; Tapan subdistrict, Sribanpod district, Phatthalung province.
